# Massive Tricuspid Regurgitation After Implantable Cardioverter Defibrillator Lead Extraction: A Late Complication From Ghost Material

**DOI:** 10.1016/j.jaccas.2025.105292

**Published:** 2025-10-29

**Authors:** Sarah Atighetchi, Fabien Praz, Fabian Noti, David Reineke, Caroline Chong-Nguyen

**Affiliations:** a Department of General Internal Medicine, Inselspital, Bern University Hospital, University of Bern, Bern, Switzerland; b Institute of Primary Health Care (BIHAM), University of Bern, Bern, Switzerland; c Department of Cardiology, Inselspital, Bern University Hospital, University of Bern, Bern, Switzerland; d Department of Cardiac Surgery, Inselspital, Bern University Hospital, University of Bern, Bern, Switzerland; e Cardiology, Hospital Center of Biel, Biel, Switzerland

**Keywords:** transthoracic and transesophageal echocardiography, transvenous lead extraction, tricuspid regurgitation, tricuspid valve

## Abstract

**Background:**

Moderate tricuspid regurgitation (TR) is seen as pathological and can result from atrial or ventricular dilatation or interaction with device leads. Rarely, fibrotic remnants after transvenous lead extraction (TLE), referred to as “ghost material”, can impair tricuspid valve function.

**Case Summary:**

A 64-year-old woman presented with dyspnea and fatigue. Echocardiography revealed massive primary TR, with a flail-like appearance of the posterior leaflet caused by a linear mobile structure retracting the leaflet. During surgery, the structure was identified as remnant fibrotic material of previous TLE, causing progressive massive TR in the course of 20 years.

**Discussion:**

After TLE, residual fibrotic remnants, referred to as “ghost material”, when attached to the valve apparatus can lead to progressive TR, even after many years.

**Take-Home Message:**

Long-term outpatient follow-up is essential after TLE, as TR may progress and “ghost material” may even further induce fibrosis on the valve apparatus.

## Graphical Abstract

**Figure:**
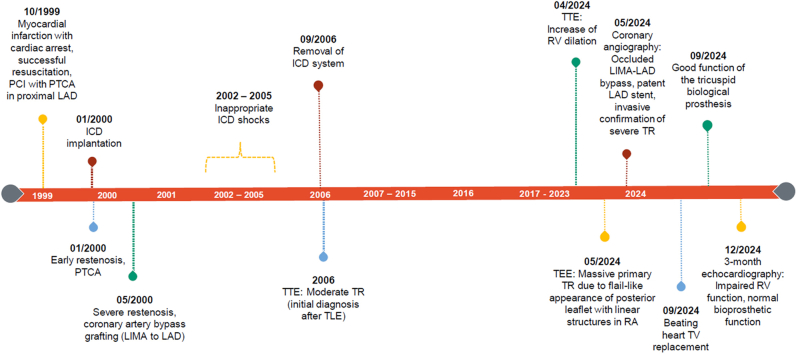

## History of Presentation

We report a rare case of progressive worsening leading to massive tricuspid regurgitation (TR) 18 years after transvenous lead extraction (TLE), caused by persistent “ghost material” fused to the tricuspid leaflets.

**Take-Home Messages**

• This case highlights a rare, long-term complication of implantable cardioverter-defibrillator lead extraction, manifesting nearly 2 decades later as massive tricuspid regurgitation due to an unusual “ghost” lead attachment.
• Long-term outpatient follow-up in patients with a history of device implantation and/or extraction is warranted with careful monitoring of tricuspid valve function.

A 64-year-old woman presented with progressive dyspnea (NYHA functional class II) and fatigue during daily activities. Electrocardiogram showed atrial fibrillation. Laboratory values where within normal range.

**Visual Summary:**
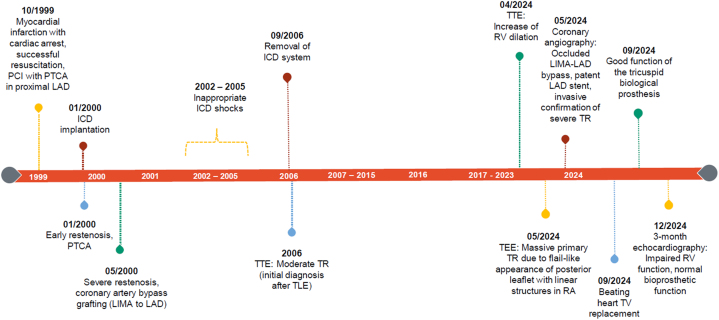
Timeline of Medical History The figure shows all important cardiac milestones related with the development, the diagnosis, and the therapy of the massive tricuspid regurgitation due to “ghost material”.

**Figure 1:**
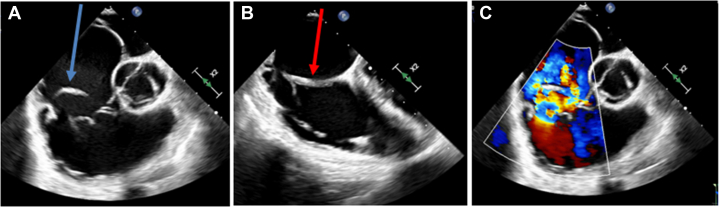
Transesophageal Echocardiography Focused on the Tricuspid Valve (A) Aspect of posterior tricuspid leaflet flail (blue arrow). (B) Aspect of “ghost material” (red arrow) looking like “aberrant” chordae attached from the lateral wall of the right atrium to the tricuspid valve. (C) Massive tricuspid regurgitation with eccentric jet.

**Figure 2:**
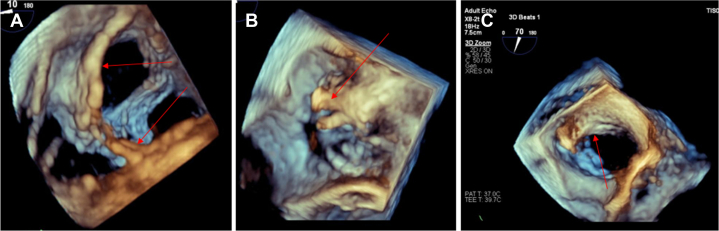
Transesophageal Echocardiography With 3D Reconstruction Focused on the Tricuspid Valve (A) Right ventricular view with insertion of the fibrous tissue (“ghost material”) on the tricuspid valve (red arrows). (B and C) Right atrial view with insertion of the fibrous tissue (“ghost material”) on the tricuspid valve and the atrial wall (red arrow).

Transthoracic and transesophageal echocardiography (TTE and TEE) showed massive primary TR (PISA 2D ERO 72 mm^2^, right ventricle [RV] 42 mL, vena contracta 16 mm, peak of velocity 1,96 m/s) due to a flail-like appearance of the posterior leaflet (Figures 1A and 2). The posterior leaflet appeared thickened and retracted, while suspended into the right atrium by linear structures resembling aberrant chordae or a supporting structure attached to the lateral wall of the right atrium and to the tricuspid annulus (Figure 1B). This led to an eccentric and massive TR jet (Figure 1C) with severe coaptation defect. The RV was dilated (RV base 49 mm) with a paradoxical septal motion and preserved function (tricuspid annular plane systolic excursion: 17 mm, fractional area change: 55%). The left ventricular dimensions and function were within normal limits (Videos 1 and 2).

## Past Medical History

The patient had a history of myocardial infarction in 1999 with cardiac arrest and successful resuscitation. Percutaneous coronary intervention was performed, with stent implantation in the proximal left anterior descending (LAD), followed by repeat stenting for early restenosis in 2000. Due to recurrent in-stent restenosis, coronary artery bypass grafting (left internal mammary artery to LAD) was performed the same year. Primary prevention implantation of an implantable cardioverter-defibrillator (ICD) followed because of heart failure with reduced left ventricular ejection fraction of 25%. The patient experienced inappropriate ICD shocks in 2002 due to lead failure. The single-coil ICD lead was interventionally extracted, and a new single-coil ICD lead was implanted. Post TLE, a moderate, eccentric TR with fibrotic remnants in the right atrium was documented (Videos 3 and 4). In 2005, the patient again suffered from inappropriate ICD discharges, necessitating TLE and lead replacement. One year later, the Medtronic sprint fidelis single-coil lead presented sensing issues. Given the recurrence of ICD system complications and the absence of arrhythmic events as well as a now normalized ventricular function, the ICD system was removed in 2006.

## Differential Diagnosis

Considering the medical history with TLE, a retained insulation lead fragment leading to progressive fibrosis and a potential congenital anomaly, although unlikely, are possible factors regarding the etiology of the TR. A floating structure was observed on the posterior leaflet, which could be interpreted as endocarditis in the differential diagnosis. However, in the absence of febrile symptoms and with normal laboratory parameters, we considered this unlikely.

## Investigations and Management

Taking into account patient's comorbidities, previous cardiac surgery, and anatomical features, an interdisciplinary team evaluated surgical and interventional options. Due to extensive structural deformation of the tricuspid valve (TV) with suspension of the posterior leaflet, percutaneous repair or replacement was deemed unfeasible. The TRISCORE was 0/12 (1% of mortality for isolated TV surgery).^1^ The heart team decided to proceed with surgical reoperation via median resternotomy.

A preoperative coronary angiography revealed the left internal mammary artery-LAD bypass to be occluded with a patent LAD stent. In addition, massive TR was invasively confirmed with a pathological right atrial v-wave of 16 mm Hg.

**Figure 3:**
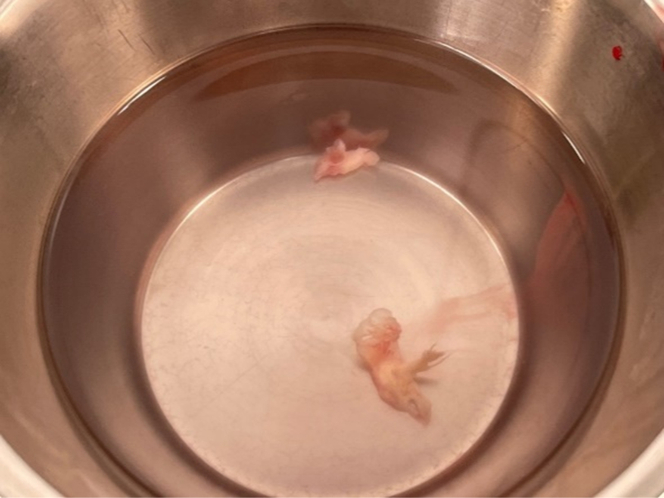
“Ghost Material” During surgical extraction, the remnants were identified as “ghost material”.

Intraoperatively, structures in the right atrium appeared to exert tension on the posterior tricuspid leaflet. Initially suspected to be aberrant chordae or supporting structures, the tissue clearly originated from fibrotic remnants, known as “ghost material” (Figure 3). Based on the surgical team's experience and intraoperative findings, the residual tissue was most likely consistent with “ghost material”. Consequently, histological processing of the material was not performed. The “ghost material” had, along the initial course of the later extracted ICD lead, fused with the posterior leaflet and the lateral right atrial wall. This caused the leaflet to have a flail-like appearance and resulted in massive TR worsened by dilation of the right chambers. A beating heart TV replacement was performed with a 27-mm Edwards Mitris bioprosthesis. Intraoperative TEE confirmed good prosthetic valve function without paravalvular leakage.

On the second postoperative day, the patient presented with hypotension and tachycardia due to hemopericardium, which was evacuated with instantaneous hemodynamic stabilization. The following postoperative course went uneventful.

## Outcome and Follow-Up

Patient symptoms improved after surgery, in particular dyspnea. The postoperative and 3-month transthoracic echocardiography revealed normal left ventricular function, with impaired RV function, and normal bioprosthetic function (mean Gr. 3 mm Hg) in sinus rhythm.

## Discussion

After TLE, residual fibrotic remnants, often referred to as “ghost material”, when attached to the valve apparatus, can lead to progressive TR, even many years after TLE.

To clarify the pathophysiological aspects further, mechanistic studies including observations during autopsy and surgery showed leaflet perforation, entanglement, impingement, fibrosis, adherence, or injury during TLE as potential causes.^2^^,^^3^

The development of fibrotic tissue encapsulating cardiac implantable electronic device leads with tissue remnants following TLE has been previously reported^3^ and may result from tissue overgrowth and mechanical interaction with lead encapsulation due to thrombosis followed by collagenous fibrosis, as well as endothelialization of the fibrous capsule.^4^^,^^5^ This process can further result into a fusion and adherence of the endocardial lead to the TV leaflets, chordae, and papillary muscles with worsening TR.^3^ Intracardiac excess tissue, so called “ghosts”, has been reported in 8% of patients following TLE in retrospective studies and in 14% of patients in prospective studies.^4^, ^5^, ^6^, ^7^ There are 2 different categories of “ghosts”: stable “ghosts” that are attached to the cardiovascular wall, and flying “ghosts” consisting of freely moving fibrous tissue.^8^

Few studies have investigated the prognostic implications and mortality associated with the presence of “ghosts”.

Narducci et al^4^ identified the presence of “ghosts” as an independent predictor of mortality after TLE due to device- or lead-related infections. Similarly, Le Dolley et al^9^ and Diemberger et al^10^ linked “ghosts” to device infection and poor prognosis, especially when detected by TEE in combination with a closed cardiac implantable electronic device pocket. Recent studies have questioned the clinical relevance of “ghosts”, whereas Nowosielecka et al^5^ identified lead number and dwell time as key predictors of their formation.

Interaction between a TV leaflet and “ghost material” is a rare complication that has not been reported so far, particularly late after TLE. This emphasizes the necessity of long-term outpatient follow-up of TV function after TLE and raises the question whether lead extraction achieves the desired effect in certain morphologies like suspension of a leaflet into the atrium.

## Conclusions

This case report describes a rare, late complication of TLE, manifesting with right heart failure nearly 2 decades after TLE due to suspension of the posterior TV leaflet through “ghost material”. Although remnant fibrous tissue following TLE is frequently observed, its involvement in the occurrence of massive TR has not been described. Long-term outpatient follow-up in patients with a history of device implantation and/or extraction is warranted with careful monitoring of TV function.

**Equipment List:** Diagnosis and Treatment of Massive Tricuspid Regurgitation

Imaging	• Transthoracic and transesophageal echocardiography (TTE and TEE) (Philips)Healthcare, USA) ◦ Philips EPIQ ◦ Philips X8-2t TEE probe
Surgical equipment	• Valve replacement (Edwards Lifesciences, USA) ◦ 27-mm Edwards MITRIS RESILIA mitral valve

## Funding Support and Author Disclosures

The authors have reported that they have no relationships relevant to the contents of this paper to disclose.

## Appendix

Video 1 Transthoracic Echocardiography Before Tricuspid Valve Surgery Dilatation of right heart chambers. Fibrous tissue attached to the tricuspid leaflets.

Video 2 Transthoracic Echocardiography With Color Doppler Before Tricuspid Valve Surgery Massive tricuspid regurgitation.

Video 3 Transthoracic Echocardiography After Transvenous Lead Extraction Tricuspid valve and the attachment from the fibrous tissue to the leaflets.

Video 4 Transthoracic Echocardiography With Color Doppler After Transvenous Lead Extraction Moderate tricuspid regurgitation.

## References

[1] Dreyfus J., Audureau E., Bohbot Y. (2022). TRI-SCORE: a new risk score for in-hospital mortality prediction after isolated tricuspid valve surgery. Eur Heart J.

[2] Praz F., Muraru D., Kreidel F. (2021). Transcatheter treatment for tricuspid valve disease. EuroIntervention.

[3] Caiati C., Luzzi G., Pollice P., Favale S., Lepera M.E. (2020). A novel clinical perspective on new masses after lead extraction (ghosts) by means of intracardiac echocardiography. J Clin Med.

[4] Narducci M.L., Di Monaco A., Pelargonio G. (2017). Presence of ‘ghosts’ and mortality after transvenous lead extraction. EP Eur.

[5] Nowosielecka D., Jacheć W., Polewczyk A., Tułecki Ł., Stefańczyk P., Kutarski A. (2022). “Ghost”, a well-known but not fully explained echocardiographic finding during transvenous lead extraction: clinical significance. Int J Environ Res Public Health.

[6] Deharo J.C., Dreyfus J., Bongiorni M.G. (2025). Management of patients with transvalvular right ventricular leads undergoing transcatheter tricuspid valve interventions: a scientific statement of the European Heart Rhythm Association and the European Association of Percutaneous Cardiovascular Interventions of the ESC endorsed by the Heart Rhythm Society, the Asian Pacific Heart Rhythm Society and the Canadian Heart Rhythm Society. Europace.

[7] Andreas M., Burri H., Praz F. (2023). Tricuspid valve disease and cardiac implantable electronic devices. Eur Heart J.

[8] Li Y., Jiang L., Wang L., Han Q., Yin X., Feng Y. (2023). Ghost in the right atrium: a case report on successful identification of residual fibrous tissue. Heliyon.

[9] Dolley Y.L., Thuny F., Mancini J. (2010). Diagnosis of cardiac device–related infective endocarditis after device removal. JACC Cardiovasc Imaging.

[10] Diemberger I., Biffi M., Lorenzetti S. (2018). Predictors of long-term survival free from relapses after extraction of infected CIED. Europace.

